# Active nuclear import of the deacetylase Sirtuin-2 is controlled by its C-terminus and importins

**DOI:** 10.1038/s41598-020-58397-6

**Published:** 2020-02-10

**Authors:** Matthew J. G. Eldridge, Jorge M. Pereira, Francis Impens, Mélanie A. Hamon

**Affiliations:** 1 Pasteur, Chromatine et Infection G5, Paris, France; 2 Institut Pasteur, Unité des Interactions Bactéries-Cellules, Paris, France; 3 Present Address: Laboratory of Molecular Microbiology, Global Health Institute, School of Life Sciences, Station 19, EPFL-SV-UPBLO, Ecole Polytechnique Fédérale de Lausanne (EPFL), 1015, Lausanne, Switzerland; 4 Present Address: VIB Center for Medical Biotechnology, Department of Biomolecular Medicine, Ghent University, Ghent, Belgium

**Keywords:** Mass spectrometry, Protein translocation

## Abstract

The NAD-dependent deacetylase Sirtuin-2 (SIRT2) functions in diverse cellular processes including the cell cycle, metabolism, and has important roles in tumorigenesis and bacterial infection. SIRT2 predominantly resides in the cytoplasm but can also function in the nucleus. Consequently, SIRT2 localisation and its interacting partners may greatly impact its function and need to be defined more clearly. In this study we used mass spectrometry to determine the interactomes of SIRT2 in whole cells and in specific cellular fractions; cytoplasm, nucleus and chromatin. Using this approach, we identified novel interacting partners of SIRT2. These included a number of proteins that function in nuclear import. We show that multiple importins interact with and contribute to the basal nuclear shuttling of SIRT2 and that one of these, IPO7 is required for SIRT2 mediated H3K18 deacetylation in response to bacterial infection. Furthermore, we reveal that the unstructured C-terminus of SIRT2 negatively regulates importin-binding and nuclear transport. This study demonstrates that SIRT2 is actively transported into the nucleus via a process regulated by its C-terminus and provides a resource of SIRT2 interacting partners.

## Introduction

The Sirtuin family of NAD-dependent deacetylases consists of 7 members (SIRT1-7) which play key protective roles in age-related diseases and act as metabolic-stress response regulators^1^. Despite sharing conserved NAD-binding and catalytic domains Sirtuins have diverse roles across multiple subcellular compartments. SIRT1, 6 and 7 are primarily nuclear proteins; SIRT3, 4 and 5 localise to the mitochondria and SIRT2 is the only Sirtuin which predominantly resides in the cytoplasm^1^. These differences are in large part due to the distinct N- and/or C-terminal extensions of different Sirtuins^2^. These regions can regulate substrate binding; catalytic activity; contain specialised domains such as nuclear-localisation signals (NLSs), nuclear-export signals (NESs) and mitochondrial-targeting sequences (MTSs) which control subcellular localisation; and serve as platforms for the addition of post-translational modifications (PTMs) which adjust Sirtuin function^3–5^.

SIRT2 has been studied primarily for its roles within the cytoplasmic milieu where it was first identified as a tubulin deacetylase^6^. It has since been demonstrated to have regulatory roles during oxidative stress and inflammatory responses via the direct deacetylation of FOXO3 and NF-κB respectively, as well as multiple pathways relating to glucose and lipid metabolism^7^. The functionality of SIRT2 is extended further by its capacity to localise to different cellular compartments including the ER-Golgi intermediate compartment (ERGIC)^8^, mitochondria^9,10^ and notably the nucleus and chromatin^11–13^. Despite having a predominantly cytoplasmic localisation, SIRT2 is in fact continuously shuttled between the cytosol and the nuclear compartment^11^. During various physiological conditions, such as mitosis^6,14^ and *Listeria monocytogenes* infection^12,13^, SIRT2 accumulates in the nucleus and mediates the deacetylation of H4 lysine 16 and H3 lysine 18 respectively. Furthermore, SIRT2 regulates non-histone nuclear proteins such as p300 and p53, which have been identified as bona fide interacting partners and substrates of SIRT2^15,16^. However, despite a well characterised export mechanism which requires the exportin CRM1, the machinery and mechanisms which underlie SIRT2 nuclear import are unknown^11^.

One important factor contributing to SIRT2 localisation and function is the differential splicing of SIRT2 RNA which produces distinct isoforms with varying N- or C- terminal extensions^17,18^. These changes alter the presence of specific functional domains and PTMs, creating SIRT2 variants with distinct roles^12,18^. For instance, isoform 2 is able to shuttle to the nucleus but lacks the first 37 amino acids which are required for chromatin-association^12^. Isoform 5 of SIRT2 constitutively localises to the nucleus as it lacks amino acids 6–76 which contain the NES (amino acids 41–51). In fact, isoform 5 displays no catalytic activity towards synthetic substrates or known protein substrates such as histones H3 or H4 and is thought to have non-enzymatic roles in the nucleus^18^. In addition, SIRT2 isoforms are heterogeneously expressed across different tissues. In skeletal muscle the full-length isoform 1 is the most abundant form of SIRT2, whereas isoform 2 is more prevalent in brain and spinal cord tissues. In other tissues such as heart, liver and kidney the two isoforms are equally expressed^17^. Irrespective of the isoform, maintaining appropriate SIRT2 functionality is critical for preserving cell homeostasis. Dysregulation of SIRT2 activity, abundance or nuclear levels have been associated with poor cancer prognosis and heightened metastasis^19,20^. However, the molecular mechanisms that control and maintain appropriate SIRT2 function, for instance its substrate specificity and localisation, remain unknown.

We employed a proteomics-based approach to identify SIRT2-interacting partners which may act as substrates or regulators of SIRT2. Using this approach, we generated an interactome of 449 proteins which contains more than 200 previously unidentified putative SIRT2-interacting partners. Amongst them we found that proteins involved in nuclear transport are significantly enriched. Further exploration confirmed that SIRT2 interacts with multiple nuclear importin proteins which contributes to the basal nuclear shuttling of SIRT2. We further show that blocking nuclear import through inactivation of importins limits the function of SIRT2 towards H3K18. Additionally, we reveal that the unstructured C-terminus acts as a negative regulator of nuclear import by limiting importin-SIRT2 interactions.

## Results

### Whole cell interactome reveals new putative SIRT2 interacting partners

**Figure 1:**
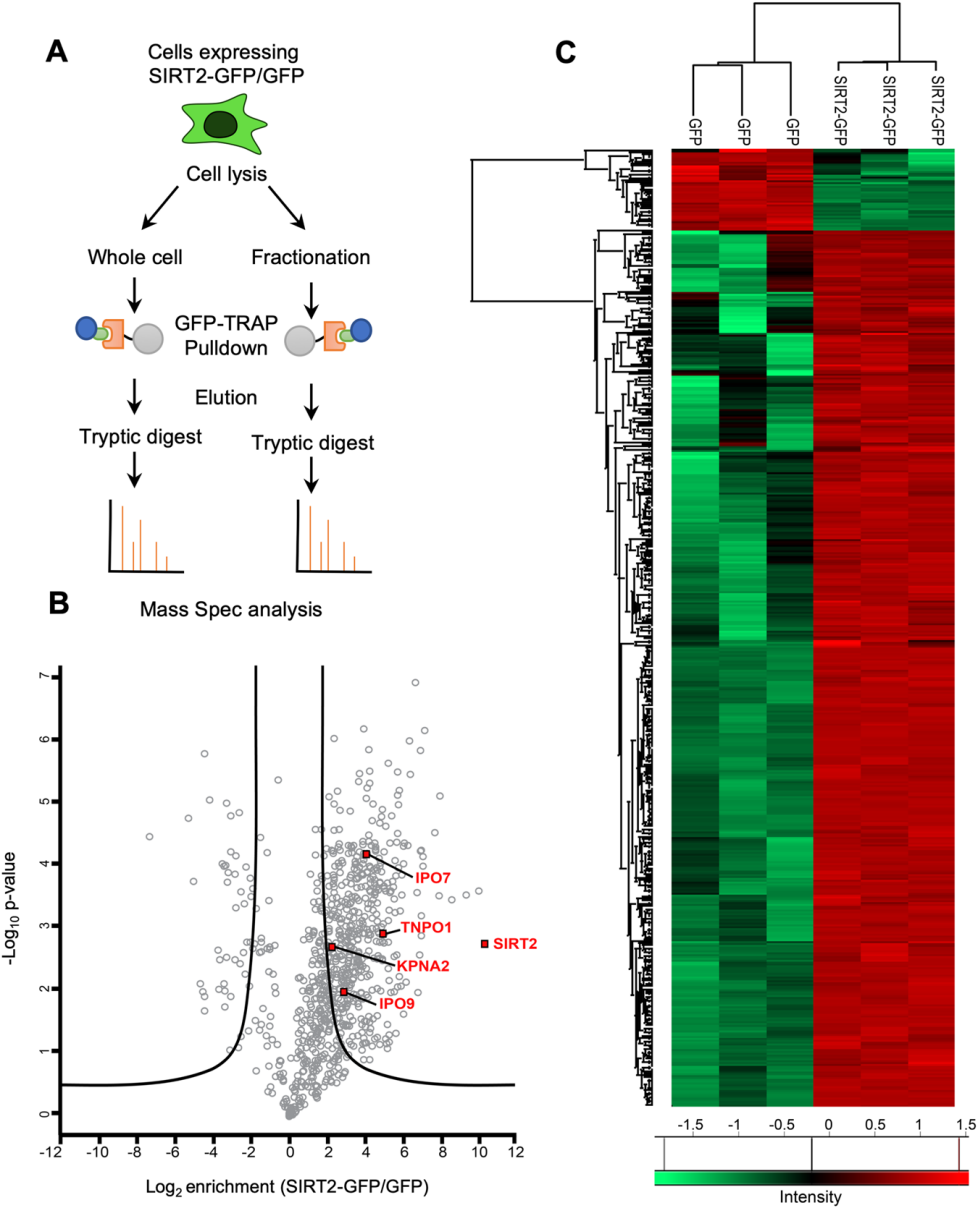
Characterisation of SIRT2 whole-cell interactome **(A)** Workflow of differential whole cell and fractionation co-immunoprecipitations of SIRT2-GFP for the identification of interaction partners by mass spectrometry. **(B)** Volcano plot of interactome results showing proteins that are significantly enriched by SIRT2-GFP immunoprecipitation. Significant hits were determined by t-testing using an FDR of 0.01 and S0 value of 2. Proteins involved in nuclear import are highlighted. **(C)** Heatmap depicting the Z-scored intensities of all significant proteins hits from (B) after non-supervised hierarchical clustering. The heatmap includes the 449 SIRT-GFP binders and 41 GFP binders.

To identify interacting partners of SIRT2 (isoform 1), HeLa cells were transfected with either GFP alone or SIRT2-tagged at its C-terminus with GFP (SIRT2-GFP). We conducted cell lysis using RIPA buffer to maximise the rupture of cellular organelles, particularly the nucleus, and release of membrane associated proteins. SIRT2-GFP or GFP alone were then immunoprecipitated using GFP-Trap® agarose beads. Extracted proteins were eluted and analysed by LC-MS/MS to indentify putative SIRT2-interacting proteins. To gain further insight into the localisation of specific SIRT2 interactions, the same approach was applied to cell lysates which had been fractionated into cytosolic, nuclear soluble and chromatin fractions (Fig. 1A).

Mass spectrometry (MS) analysis from whole cell lysates identified over 600 proteins which co-immunoprecipitated with SIRT2-GFP (Table S1). Statistical analysis by t-testing using a false discovery rate of 0.01 and S0 value of 2 showed that approximately 450 of the identified proteins were significantly enriched by SIRT2-GFP over the GFP alone immunoprecipitation control (Fig. 1B and Table S1). Heat-map analysis of the Z-scored intensities of identified peptides further highlights the extent of protein enrichment by SIRT2-GFP over GFP alone (Fig. 1C). Identified peptides which were common between the whole cell and specific cellular fractions were used to illustrate the compartments of the cell in which these interactions may occur (Table S1). We identified established SIRT2 interaction partners such as Exportin-1, EP300, CBP and the archetypal substrate of SIRT2, α-tubulin. Interestingly, we identified more than 200 proteins which have not previously been reported as SIRT2 interactors.

To gain further insight into the functional roles of the newly identified SIRT2 interactors, gene ontology term enrichment analysis was performed using PANTHER^21^. The most enriched GO terms for biological processes included tricarboxylic acid cycle^22^, vesicle-mediated transport^8^ and fatty acid catabolic processes^23^ (Table S2). These are consistent with the growing body of reports which implicate SIRT2 as a key regulator of metabolic processes within the cell^7^.

Among the significantly enriched putative interactors which had not previously been reported to interact with SIRT2 were nuclear transport proteins, notably the importins; Importin subunit alpha-1 (KPNA2),, Importin 7 (IPO7), Transportin 1 (TNPO1) and Importin 9 (IPO9) (Fig. 1D) which recognise and bind cargo proteins prior to their transport into the nuclear compartment. Because the mechanism regulating SIRT2 nuclear import had not yet been characterised, we focused our analysis on the importins TNPO1, IPO7 and the importin adaptor KPNA2 as these proteins are responsible for cargo recognition and binding.

### SIRT2-importin interactions

**Figure 2:**
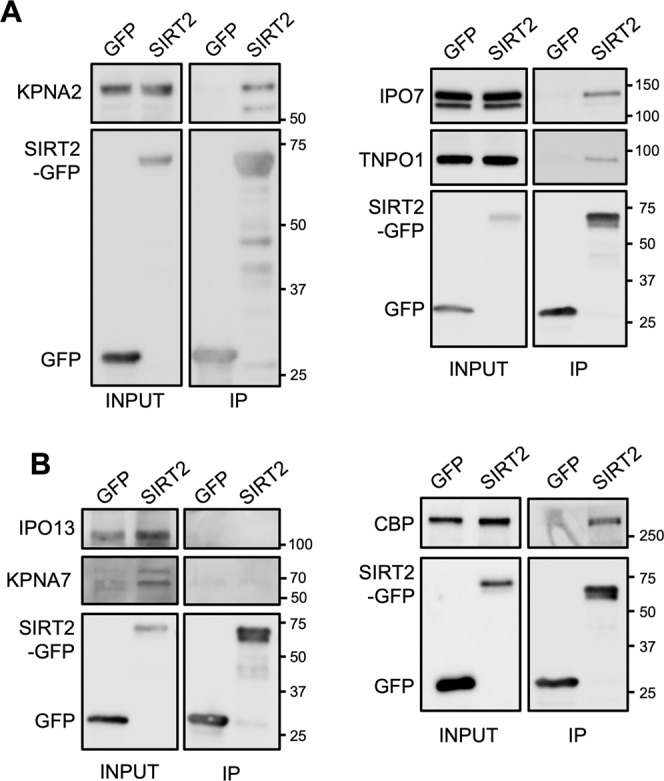
SIRT2 interacts with multiple importins. SIRT2-GFP or GFP alone where immunoprecipitated using GFP-Trap® agarose beads for 1 hr. Cell lysates (INPUT) and IP fractions were immunoblotted using antibodies against GFP and **(A)** KPNA2, IPO7, TNPO1 or, **(B)** IPO13 and CBP which served as controls. Images are representative of 3 independent experiments. Uncropped blots are presented in Supplementary S3.

To validate the interactions between SIRT2 and the identified importins, GFP alone and SIRT2-GFP were immunoprecipitated from transfected HeLa cells and analysed by Western blot. Endogenous KPNA2, IPO7 and TNPO1 proteins co-immunoprecipitated with SIRT2-GFP and were noticeably enriched as compared with the GFP alone control (Fig. 2A). These interactions were also detected in immunoprecipitations from HEK293T cells, suggesting that SIRT2-importin complexes are likely common to multiple cell types (Fig. S2A). Immunoprecipitation of SIRT2 which had a N-terminal mCherry tag also co-purified with KNPA2, IPO7 and TNPO1, ensuring that the type of tag and its position did not affect these interactions (Fig. S2B). To verify the specificity of these interactions we also carried out western blot analyses on IPO13 and KPNA7 which were not identified in our interactome and represent both families of nuclear-cargo receptors. As expected IPO13 and KPNA7 were not detected by western blot in SIRT2-GFP immunoprecipitation samples. As a control, we also tested for the presence of CREB-binding protein (CBP), a previously reported substrate and binding partner of SIRT2 that was also present in our interactome. Western blot analysis confirmed co-immunoprecipitation (CO-IP) of CBP with SIRT2-GFP (Fig. 2B). By CO-IP we therefore validated the MS results that the nuclear import proteins KPNA2, TNPO1 and IPO7 interact with SIRT2.

### RNAi silencing of importins dampens nuclear accumulation of SIRT2

The cytoplasmic localisation of SIRT2 is maintained by the exportin CRM1, which shuttles SIRT2 out of the nucleus^11^. To study nuclear import, we blocked nuclear export with Leptomycin B (LMB), a potent inhibitor of CRM1-mediated export, as it has previously been reported to cause SIRT2 localisation and accumulation in the nucleus. To investigate whether the identified importins contributed to the basal nuclear import we performed RNAi of KPNA2, IPO7 and TNPO1 and measured the amount of SIRT2 nuclear accumulation in response to LMB. We first generated a HeLa cell line stably expressing SIRT2-GFP to enable us to monitor homogeneous fluorescence across the cell population. SIRT2-GFP cells were transfected with siRNAs targeting either KPNA2; IPO7; TNPO1; a combination of all three (ALL) (Fig. S1B). KPNA7, which was not identified in our interactome was used as a negative control. LMB was then used to induce the nuclear accumulation of SIRT2.

**Figure 3:**
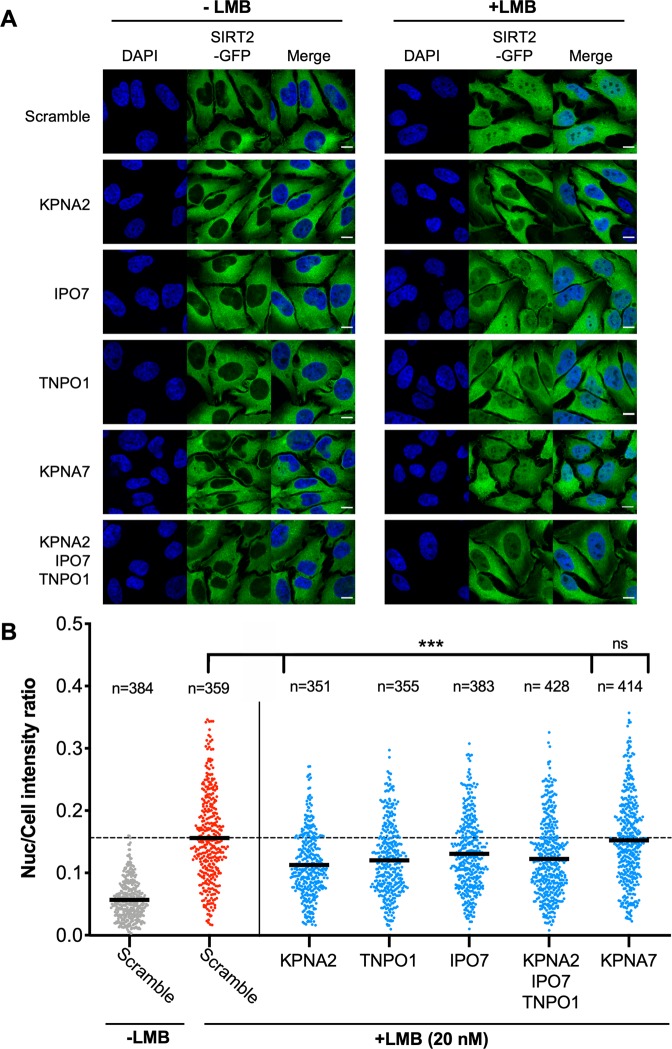
Importins KPNA2, TNPO1 and IPO7 mediated basal nuclear import of SIRT2. **(A)** Images of HeLa cells stably expressing SIRT-GFP (green) and transfected with stated siRNAs were left untreated or treated with LMB (20 nM) for 1 hour. Nuclei (blue) were stained using DAPI. Scale bar, 10 µm. **(B)** Graphs show nuclear: whole cell intensity ratio of SIRT2-GFP. Each data point represents a single cell measured from 3 independent experiments. Statistical significance was determined between importin siRNA treated (blue) and scrambled siRNA cells (red) treated with LMB as determined by one-way ANOVA with Dunnet correction for multiple comparisons ***p < 0.0001.

In untreated (-LMB) cells transfected with scramble or importin targeting siRNAs, SIRT2 predominately remains localised to the cytoplasm and is strongly occluded from the nucleus (Fig. 3A). Quantification of SIRT2-GFP fluorescence intensity yielded an average nuclear/total cell ratio of approximately 0.05 (Figs. 3B and S1A). In scrambled siRNA treated cells, the addition of LMB (20 nM) for 1 hour caused SIRT2 to accumulate in the nucleus (Fig. 3A) raising the average nuclear intensity ratio to 0.156 (Fig. 3B). Silencing the expression of either KPNA2, TNPO1 or IPO7 (Fig. S1B) significantly reduced the import of SIRT2-GFP to the nucleus as detected by fluorescence microscopy (Fig. 3A,B). In contrast, silencing the expression of the non-interacting importin KPNA7 did not affect nuclear accumulation of SIRT2-GFP (Fig. 3A,B). Interestingly, combined transfection of KPNA2, TNPO1 and IPO7 siRNAs reduced nuclear accumulation to a level comparable to that of an individually silenced importin. Such results suggest that these proteins may act as a complex where each member is essential for proper function. Together our results show that the basal import of SIRT2 into the nucleus is jointly regulated by the KPNA2, IPO7 and TNPO1 importins.

### The C-terminus of SIRT2 negatively regulates importin binding and nuclear import

**Figure 4:**
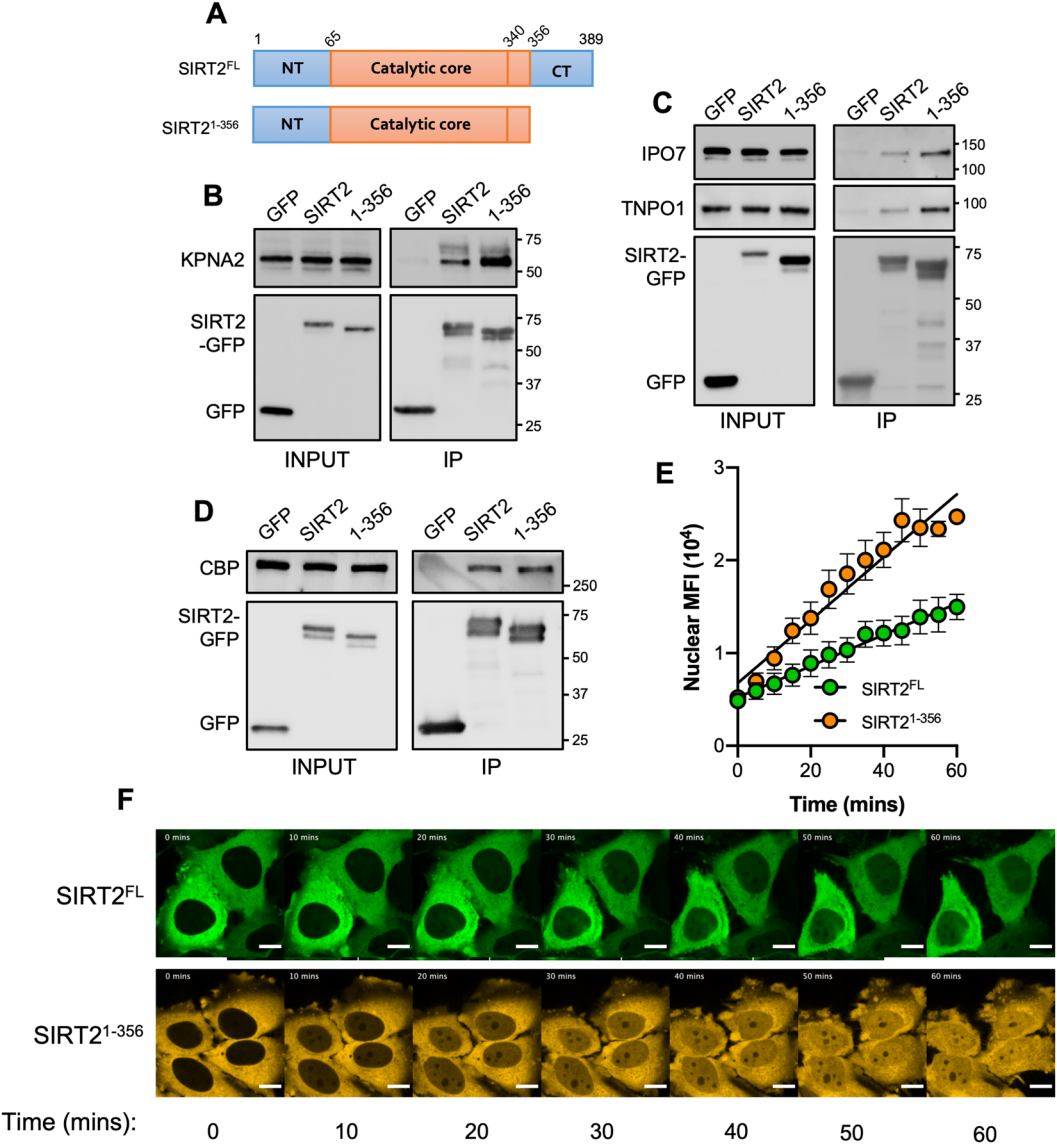
The C-terminus of SIRT2 negatively regulates importin binding and nuclear accumulation. **(A)** Schematics of full-length SIRT2 (SIRT2^FL^) and SIRT2 lacking its unstructured C-terminus (SIRT21–356). GFP, SIRT2^FL^ or truncated SIRT21–356 were immunoprecipitated for 1 hr. Cell lysates (INPUT) and IP fractions were immunoblotted for with antibodies against GFP and **(B)** KPNA2, **(C)** IPO7 and TNPO1 **(D)** CBP which served as control. Data are representative of 3 experiments. Uncropped blots are presented in Supplementary S3. **(E)** Quantification of live-cell imaging of HeLa cells expressing SIRT2^FL^ (green) or SIRT21–356 (orange) treated with LMB (20 nM). Graph shows the mean fluorescence intensity in the nucleus over time from, images were acquired every 5 mins. Error bars represent SEM of 4 independent experiments. Lines represents the slope of a regression line for each data set. **(F)** Representative still images used for analysis in (E). Scale bar, 10 µm.

A clearly defined NLS has not been identified in the primary sequence of SIRT2. *In silico* sequence analyses reveal low scoring predicted sites as non-canonical NLSs are difficult to predict. For other nuclear Sirtuins (SIRT1, SIRT6 and SIRT7) the domains which regulate their localisation reside in the N- or C- terminal extensions which extend from the catalytic core ^24–27^. With regards to SIRT2 the N-terminus has been shown to mediate nuclear export and does not impact import^12,18^. Because the C-terminus of SIRT2 has an established role in regulating its catalytic activity^3,28^ we asked whether this domain could also control its localisation. We therefore generated a truncated SIRT2 (SIRT2^1–356^) which lacked the 32 amino acid, unstructured C-terminal end (amino acids 357–389) to see if this region impacted nuclear localisation (Fig. 4A).

We first tested SIRT2^1–356^ for its ability to bind the importins KPNA2, IPO7 and TNPO1. To our surprise SIRT2^1–356^ co-immunoprecipitated with heightened levels of endogenous KPNA2, IPO7 and TNPO1 when compared with the full-length protein (Fig. 4B,C) suggesting that loss of the C-terminus increases importin binding. It should be noted that the position of the fluorescent tag does not affect this process as SIRT2^1–356^ tagged at its N-terminus with mCherry also binds more strongly to importins (Fig. S2B). To assess whether this effect was the result of a general enhancement in protein binding we tested the interaction of SIRT2^1–356^ with CBP, a non-importin-interacting partner. Both the full-length and truncated protein co-immunoprecipitated similar amounts of CBP, indicating that the increased binding capacity of SIRT2^1–356^ is specific to importins (Fig. 4D).

We next tested whether the increased binding of importins to SIRT2^1–356^ would translate into increased nuclear import. In that aim we performed live-cell microscopy to track the nuclear import of full-length SIRT2 (SIRT2^FL^) or SIRT2^1–356^. In resting cells, the cellular distribution of SIRT2^1–356^ remains the same as SIRT2^FL^ and is extruded from the nucleus maintaining a net cytoplasmic localisation (Fig. 4E). However, we found that following LMB treatment SIRT2^1–356^ translocated to the nucleus more rapidly and to a greater extent than SIRT2^FL^ over 60 minutes and showing a significantly heightened slope (Fig. 4E,F, Movie S1). These data show that the nuclear import of SIRT2 is negatively regulated by its C-terminus by reducing the binding of importins KPNA2, IPO7 and TNPO1.

### Importin 7 is required for SIRT2-mediated H3K18 deacetylation during infection with *L. monocytogenes*

**Figure 5:**
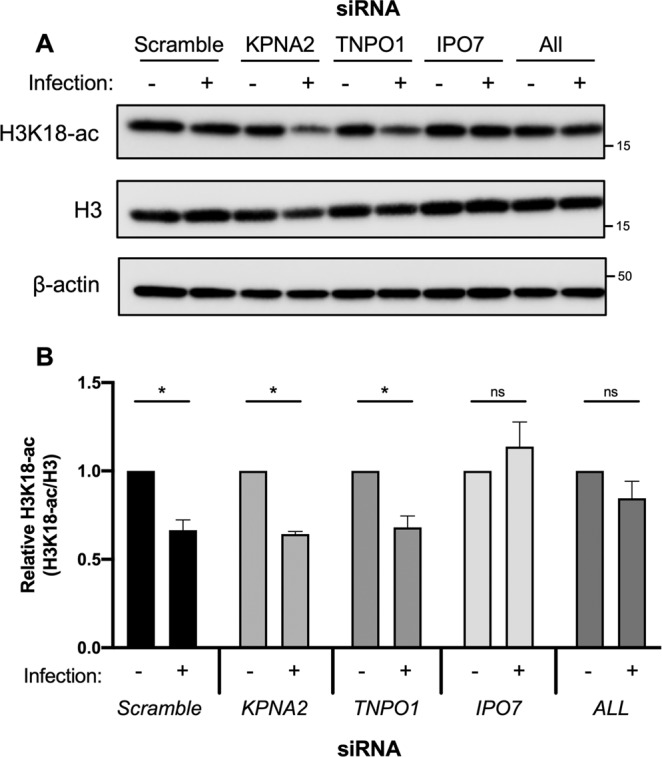
IPO7 regulates nuclear functions of SIRT2 during *L. monocytogenes* infection. HeLa cells were transfected with stated siRNAs and either left uninfected (-) or infected with *L. monocytogenes* for 6 hours a MOI 100. **(A)** Representative image of H3K18 acetylation levels detected by immunoblotting in uninfected HeLa (−) and *L. monocytogenes*–infected cells (+) transfected with stated siRNA. Uncropped blots are presented in Supplementary S3. (**B**) Quantification of H3K18 acetylation levels. Band intensity of H3K18-ac and total H3 levels are first normalised to β-actin followed by a second normalisation of H3K18-ac to total H3. Values are expressed as normalised band intensity relative to uninfected. Error bars represent SEM of 4 independent experiments. Statistical significance was calculated using two-way ANOVA with Fisher’s LSD test. *P < 0.001.

SIRT2 has been shown to accumulate in the nucleus where it acts as a histone deacetylase^13,14^. One such event which triggers SIRT2 accumulation into the nucleus is infection with the gram-positive bacterial pathogen *Listeria monocytogenes* which results in deacetylation of histone H3 at lysine-18 (H3K18)^12,13^. Our previous findings showed that dephosphorylation of SIRT2 at serine-25 is required to permit its binding to chromatin, however this played no role in nuclear import^12^. To examine whether the identified importins played a role in permitting the nuclear functions of SIRT2, we tested whether RNAi effected H3K18 deacetylation during *Listeria monocytogenes* infection. HeLa cells were transfected with siRNAs targeting KPNA2, TNPO1, IPO7 or a combination of the three (ALL) and incubated for 48 hours before being infected at a MOI of 100. Following infection HeLa cells transfected with scramble control siRNA displayed lower H3K18-ac levels and silencing the expression of either KPNA2 or TNPO1 had no effect on this process (Fig. 5A,B). Interestingly, cells silenced for IPO7 or all three importins did not undergo H3K18 deacetylation suggesting that IPO7 is specifically required to allow SIRT2 to carry out nuclear functions in response to *Listeria monocytogenes* infection.

## Discussion

In this study, we present comprehensive whole-cell and cell fraction specific interactomes for the full-length isoform 1 of SIRT2 which has identified over 200 novel putative interacting partners in HeLa cells. Our work provides a resource for the further study of SIRT2-regulated processes. Gene ontology analysis shows that SIRT2-interacting partners function within many diverse processes across the cell (Table S2). These include previously known SIRT2-regulated systems such as vesicle-mediated transport, fatty acid catabolic processes, and the tricarboxylic acid cycle which were among the most significantly enriched processes in our SIRT2 interactome^1,7^.

Many of the newly identified putative SIRT2 interactors are metabolic enzymes which reside within the mitochondria, such as fumarate hydratase and the isocitrate dehydrogenase α/β subunits. Identification of such enzymes is consistent with recent reports that SIRT2 localises to mitochondria and regulates their function^9,10^, which coupled with our data, suggests that SIRT2 may have additional uncharacterised roles in regulating metabolic processes in mitochondria.

The SIRT2 interactome also revealed multiple proteins which have not been previously reported to associate with SIRT2. One such example being proteins that mediate the import of protein cargo into the nucleus. Among these were the α-importin adapter KPNA2 and, which forms a classical importin heterodimer with the β-importin KPNB1; the β-importins IPO7, TNPO1; secondary importin interactions such as KPNB1 and the GTPase RAN which regulates the direction of nuclear transport^29^, were also detected however these were below the significance threshold. Identification of these interactions suggests that SIRT2 nuclear import is an active process mediated by the canonical nuclear import machinery. Indeed, we were able to confirm the interactions of SIRT2 with KPNA2, IPO7 and TNPO1 by co-immunoprecipitation and Western blot, and went onto show that silencing the expression of these importins reduces the nuclear accumulation of SIRT2 following treatment with LMB. Combined silencing of all three importins did not have a cumulative effect suggesting that they may work in concert with one another. KPNA2 mediated import requires the formation of a heterodimer with the β-importin KPNB1, whereas IPO7 and TNPO1 are capable of mediating autonomous cargo import. There have been reports that IPO7 can also form functional heterodimers with KPNB1^30^. It is therefore possible that these importins may be able to form non-typical heterodimers which mediate the import of SIRT2. We also observe that SIRT2 nuclear accumulation was not completely blocked by RNAi of the three importins suggesting that SIRT2 may be shuttled into the nucleus by non-importin interactors via a so called “piggy back” mechanism^31^. Indeed, we have also identified a significant number of nuclear proteins which may contribute to this effect.

KPNA2, TNPO1 and IPO7 recognise distinct NLSs^29,32,33^. KPNA2 recognises “classical” NLSs which have SV40 large T-antigen and nucleoplasmin-like sequences, but has more recently been shown to have a broader sequence specificity and can bind “non-classical” sequences such as that found in the influenza nucleoprotein^34^. TNPO1 recognises a non-classical sequence characterized by a proline-tyrosine motif called a PY-NLS^33^, while IPO7 binds a three amino acid serine-proline-serine (SPS) nuclear translocation signal^32^. *In silico* analysis and visual inspection of the SIRT2 primary sequence reveals putative “non-classical” NLSs for each of the three importins. Recent studies also suggest that the three-dimensional structure of an NLS could be as important as the sequence itself in terms of importin recognition, making NLS identification by sequence homology more complex^35^. In addition PTMs have been shown to regulate NLS functions^32,36^, which could also be the case in light of the recent report of novel SIRT2 PTMs^12^.

**Figure 6:**
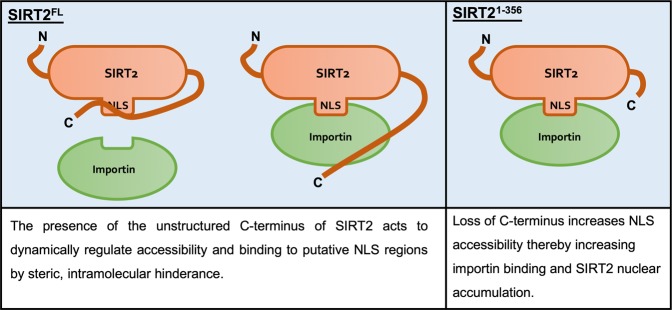
Schematic model of C-terminus mediated regulation of SIRT2 importin binding and nuclear import. In the full-length protein, the unstructured C-terminal region of SIRT2 acts as an intramolecular mask covering the putative NLSs or NLS-like regions which are recognised by importin proteins and thereby limiting their interaction. Removal of this region lifts this regulatory effect and leaves importins free to interact with and shuttle SIRT2 to the nucleus.

Though the precise NLSs of SIRT2 remain to be determined we identified its unstructured C-terminal domain as a regulator of SIRT2 nuclear import. We show that SIRT2 lacking the C-terminal unstructured region (amino acids 357–389) specifically interacts with importins more strongly. This in turn translates to increased and more rapid accumulation of SIRT2 in the nucleus following treatment with LMB. The C-terminus of SIRT2 regulates its catalytic activity by way of phosphorylation at residues S368 and S372^37,38^, and deletion of the C-terminus enhances SIRT2 catalytic activity as it is thought to act as an autoinhibitory domain^3^. We propose that the C-terminus acts in a comparable manner in the regulation of nuclear import, serving as an intramolecular mask which physically blocks importin access to the NLS or NLS-like regions of SIRT2, similar to the mechanisms described for NF-κB and NFAT4^39,40^. The loss of the C-terminus therefore allows importins to readily interact with SIRT2 causing heightened nuclear import in response to LMB (Fig. 6). Interestingly, although all three of the identified importins function in the basal nuclear shuttling of SIRT2, only IPO7 appears to function during infection with *Listeria monocytogenes*. This suggests that there may be active regulatory mechanisms which can control SIRT2 nuclear import in response to different stimuli.

Altering the localisation of SIRT2 has considerable effects on its biological activity as reports have shown that mislocalisation of SIRT2 can promote tumorigenesis and cancer progression. Increased nuclear SIRT2 has been associated with more rapid cancer relapse and death in patients with oestrogen-receptor negative breast tumours^20^, and an increase in the malignancy progression of glioblastomas^41^. Interestingly, another report found that a decrease in nuclear SIRT2 was linked with higher tumour metastasis in prostate cancer^19^. In addition, multiple cancer-associated mutations have been identified in SIRT2, however none have yet been demonstrated to affect its localisation^28^. Nine different somatic cancer-associated mutations have been identified within the C-terminal region^42^. Given our result that loss of the C-terminus heightens nuclear accumulation, it is possible that these mutations may sensitise SIRT2 to nuclear import in response to specific stimuli. Therefore, the subcellular localisation of SIRT2 has significant impacts on human health and disease progression. Our study furthers the understanding of how SIRT2 is imported into the nucleus and provides additional putative interacting partners, which could be important in health than disease.

In summary, the whole cell interactome of SIRT2 shows that it has diverse interacting partners which function in various systems across different cellular compartments. We find that multiple nuclear importins interact with SIRT2 and that KPNA2, IPO7 and TNPO1 function in its basal nuclear shuttling, which is negatively regulated by the unstructured C-terminus. Interestingly, the nature of the stimulus appears to determine which importin system is used. This reveals new proteins involved in regulating SIRT2 localisation, which coupled with its varied interacting partners emphasises the pleiotropic nature and complex activity of SIRT2.

## Experimental Procedures

### Antibodies

Antibodies used in this study are as follow; anti-GFP tag (Thermo Fisher Scientific, A-11122), anti-KPNA2 (Thermo Fisher Scientific, PA5-21034), anti-Importin 7 (Thermo Fisher Scientific, PA5-21764), anti-TNPO1 (Thermo Fisher Scientific, PA5-24581), anti-Importin 13 (Thermo Fisher Scientific, PA5-21884), anti-β-actin (Sigma, AC-15), anti-KAT3A/CBP antibody (Abcam, ab2832).

### Cell Culture and treatments

HeLa cells were maintained in minimum essential medium (MEM) plus GlutaMAX (Gibco) supplemented with 1 mM sodium pyruvate (Gibco), 0.1 mM nonessential amino acid solution (Gibco), and 10% fetal bovine serum (FBS). Cells were treated with 20 nM Leptomycin B (Sigma) in complete medium for 1 hour.

### Cloning and generation of stable cell-line

SIRT2^1–356^ (CI110) was generated by sequence- and ligation-independent cloning (SLIC)^43^ into pEGFP-N2 plasmid at the EcoRI restriction site. PCR amplification of SIRT2 (isoform 1) from pEGFP-N2-SIRT2-GFP (BUG 4186)^12^ was performed using the following primers:

Fw – CTC GAG CTC AAG CTT CGA ATT CTA TGG CAG AGC CAG ACC.

Rv – GTA CCG TCG ACT GCA GAA TTC CGA CTG GGC ATC TAT GCT G.

HeLa cells stably expressing SIRT2-GFP were generated using the Sleeping Beauty transposon system^44^. SIRT2-GFP was PCR amplified with the following primers:

Fw – ACT ACC CCA AGC TGG CCT CTG AGG CCA CCATGG CAG AGC CAG ACC.

Rv – GAT CCC CAA GCT TGG CCT GAC AGG CCT TACTTG TAC AGC TCG TC.

The resulting amplicon was cloned by SLIC into the SfiI site of pSBbi-Neo^44^, yielding pSBbi-Neo-SIRT2-GFP (CI111). HeLa were co-transfected with pSBbi-Neo-SIRT2-GFP and pCMV (CAT) T7-SB100 (expressing the SB100X transposase enzyme) and incubated for 48 hours before the addition of 0.5 mg. mL^−1^ G418 for selection.

### Co-immunoprecipitation and LC-MS/MS analysis

HeLa cells expressing SIRT2-GFP or GFP were lysed in RIPA buffer as above or fractionated as previously described^12^. The resulting fractions or lysates were split in triplicate and underwent immunoprecipitation as described below. Following the final wash immunoprecipitated proteins were eluted from GFP-Trap® beads into 200 µL of 200 mM glycine (pH 2.5). The elutes were then adjusted to pH 7.5 using 1 M Tris base and diluted with 600 µL of 50 mM ammonium bicarbonate (pH 8.0) and 2 µg trypsin (Promega). The samples were then incubated overnight for digestion at 37 °C. The resulting peptide mixture was dried and re-dissolved in 20 µL 0.1% formic acid in water/acetonitrile (98:2, v/v) of which 1 µL was injected for LC-MS/MS analysis on an EASY-nLC 1000 system (Thermo) in-line connected to a Q Exactive Plus mass spectrometer with a Nanospray Flex Ion source (Thermo). Peptides were loaded in solvent A (0.1% formic acid in water) on a reverse-phase column (made in-house, 75 µm I.D. × 300 mm, 1.9 µm beads C18 Reprosil-Pur, Dr. Maisch) and eluted by an increase in solvent B (0.1% formic acid in acetonitrile) in linear gradients from 5% to 27% in 100 minutes, then from 27% to 45% in 40 minutes and finally from 45% to 60% in 10 minutes, all at a constant flow rate of 250 nl/min. The mass spectrometer was operated in data-dependent mode, automatically switching between MS and MS/MS acquisition for the five most abundant ion peaks per MS spectrum. Full-scan MS spectra (300–1700 m/z) were acquired at a resolution of 70,000 after accumulation to a target value of 3,000,000 with a maximum fill time of 20 ms. The five most intense ions above a threshold value of 170,000 were isolated (window of 1.6 Th) for fragmentation at a normalized collision energy of 27% after filling the trap at a target value of 1,000,000 for maximum 60 ms with an underfill ratio of 1%. The S-lens RF level was set at 60 and we excluded precursor ions with single, unassigned and charge states above six from fragmentation selection.

### Protein identification and quantification

Data analysis was performed with MaxQuant (version 1.5.6.5)^45^ using the Andromeda search engine with default search settings including a false discovery rate set at 1% on both the peptide and protein level. Spectra were searched against the human proteins in the UniProt/Swiss-Prot database (database release version of August 2016 containing 20,198 human protein sequences) supplemented with the sequence of GFP. The mass tolerance for precursor and fragment ions were set to 4.5 and 20 ppm, respectively, during the main search. Enzyme specificity was set as C-terminal to arginine and lysine, also allowing cleavage at proline bonds with a maximum of two missed cleavages. Variable modifications were set to oxidation of methionine residues, acetylation of protein N-termini and phosphorylation of serine and threonine residues. Only proteins with at least one unique or razor peptide were retained and quantified by the MaxLFQ algorithm integrated in the MaxQuant software^46^. A minimum ratio count of two unique or razor peptides was required for quantification. Further data analysis was performed with the Perseus software (version 1.5.5.3)^47^ after loading the protein groups file from MaxQuant. Proteins only identified by site and reverse database hits were removed, and the LFQ protein intensity values were log2 transformed. Technical replicate samples of SIRT2-GFP and the GFP negative control sample were grouped, proteins with less than three valid values in at least one group were removed and missing values were imputed from a normal distribution around the detection limit. Then, a t-test was performed (FDR = 0.01 and S0 = 2) to compare the SIRT2-GFP sample with the GFP negative control sample and to generate the volcano plots depicted in Fig. 1B (Table S1).

### Co-immunoprecipitations

Immunoprecipitation of SIRT2-GFP and mCherry-SIRT2 was performed using GFP-Trap® and RFP-Trap® agarose beads (Chromotek) respectively, according to the manufacturer’s protocol. Briefly, 2 × 10^6^ HeLa cells were seeded in a 10 cm dish and transfected with tagged-SIRT2 or empty pEGFP-N1/pmCherry-C1. 24 hours post transfection cells were collected using PBS with 5 mM EDTA, washed once in PBS, and lysed in 200 uL RIPA buffer (10 mM Tris/Cl pH 7.5; 150 mM NaCl; 0.5 mM EDTA; 0.1% SDS; 1% deoxycholate; 1% Triton X-100) supplemented with 25U Benzonase nuclease (Millipore), 1x Complete EDTA-free protease inhibitor cocktail (Roche) and 1x PhosSTOP phosphatase inhibitor (Roche) and 1 mM PMSF. Lysates were then diluted with 600 uL of wash/dilution buffer (10 mM Tris/Cl pH 7.5; 150 mM NaCl; 0.5 mM EDTA). 40 uL was removed for input and the remaining lysate was incubated with GFP-Trap® agarose beads at 4 °C with agitation for 1 hour. The beads were washed twice in wash buffer and once in wash buffer containing 300 mM NaCl. Proteins were eluted by boiling beads in 50 uL 2x Laemmli buffer with 100 mM DTT.

### RNA interference and DNA transfections

Transient RNAi cells was carried out using ON-TARGETplus siRNAs from Dharmacon. HeLa cells were treated with siRNA for either KPNA2 (﻿SMARTpool L-004702-00-0005), IPO7 (﻿SMARTpool L-012255-00-0005), TNPO1 (﻿SMARTpool L-011308-00-0005) or KPNA7 (﻿SMARTpool L-031817-02-0005). ON-TARGETplus Non-targeting Pool siRNA (D-001810-10-05) served as the negative control. Transfections were performed using ﻿Lipofectamine RNAiMAX reagent (Invitrogen). Briefly, 3 × 10^4^ HeLa cells were seeded onto glass coverslips in a 24 well plate. 5 pmol of siRNA mixed with 1.5 µL Lipofectamine RNAiMAX in 50 µL OptiMEM (Gibco) and incubated for 10 mins then added to cells dropwise. Cells were incubated with siRNA for 48 hours before treatment. For siRNA transfections prior to bacterial infection cells were reverse transfected in 12-well plates. Briefly, 1 × 10^5^ HeLa were added to wells containing 10 pmol of siRNA mixed with 1.5 µL Lipofectamine RNAiMAX in 100 µL OptiMEM (Gibco). Cells were incubated with the transfection complexes for 48 hours prior to infection.

For transient plasmid expression HeLa cells were transfected using Lipofectamine LTX reagent. Briefly, 300 ng of plasmid DNA was diluted in 50 µL OptiMEM mixed with 0.5 µL PLUS reagent and 1.25 µL of LTX reagent. The transfection mixture was incubated for 15 mins prior to its addition onto cells. Cells were treated with DNA-Lipofectamine complexes for 4 hours; culture medium was then replaced with fresh complete MEM medium and incubated for 20 hours prior to further treatment.

### Immunofluorescences microscopy

For immunofluorescence HeLa cells were plated onto coverslips prior to treatments. Following treatments cells were washed three times in PBS and fixed using 4% PFA for 10 mins. Cells were then permeabilised for 10 mins in 0.1% Trition X-100 PBS. Nuclei were stained with 300 nM (100 ng.mL^−1^) DAPI for 15 mins, then washed three times in PBS before being mounted using Fluoromount-G® Mounting Medium (INTERCHIM). For live imaging HeLa cells were plated onto 35 mm glass bottom dishes (MatTek, P35G-1.5-10-C) in FluoroBrite™ DMEM (Gibco) supplemented with 10% FCS. Cells were treated with 20 nM LMB ﻿and images were acquired every 5 mins in an atmospheric chamber at 37 °C. All images were acquired using a Zeiss Axio Observer spinning-disk confocal microscope equipped driven by the MetaMorph software. For quantification a minimum of ten fields of view were obtained per condition of each biological replicate and the nuclear to whole cell ratio of SIRT2-GFP was determined for individual cells. Image analysis of nuclear translocation following siRNA transfections was carried out using CellProfiler^48^ and is expressed as the mean nuclear fluorescence intensity of SIRT2-GFP over the mean fluorescence of the whole cell.

### *Listeria monocytogenes* infections

Prior to infection HeLa cells were transfected as described above and serum starved in MEM supplemented with 0.25% FBS for 24 hours prior to infection. *Listeria monocytogenes* EGD (BUG600) was grown overnight in BHI with shaking at 37 °C. Bacteria were then subcultured into fresh BHI and grown to mid log phase (OD600 = 0.8–1) and subsequently washed 3x in MEM with 0.25% FBS. Bacteria were then added onto cells at a MOI of 100 and incubated for 1 hour. Cells were then washed 3x in MEM with 0.25% FBS and incubated in fresh medium for 30 minutes prior to the addition of 10 µg.mL^−1^ gentamicin for the remainder of the infection, totalling 6 hours post inoculation.

## Supplementary information

Supplemental Information.

Table S1.

Table S2.

Movie S1.

## Data Availability

The mass spectrometry proteomics data have been deposited to the ProteomeXchange Consortium via the PRIDE^49^ partner repository with the dataset identifier PXD011523.
