# Supplementary material for: Active nuclear import of the deacetylase Sirtuin-2 is controlled by its C-terminus and importins
Source: Sci Rep. 2020 Feb 10;10:2034. doi: 10.1038/s41598-020-58397-6 (PMC7010746; doi:10.1038/s41598-020-58397-6)

Supplemental Information

Active nuclear import of the deacetylase Sirtuin-2 is controlled by its C-terminus and importins

Matthew J.G. Eldridge[1,2,§], Jorge M. Pereira[1,2,3#,§], Francis Impens[2,4#], and Mélanie A. Hamon[1,*]

From the [1]Pasteur, Chromatine et Infection G5, Paris, France; [2]Institut Pasteur, Unité des Interactions Bactéries-Cellules, Paris, France; [3]Laboratory of Molecular Microbiology, Global Health Institute, School of Life Sciences, Station 19, EPFL-SV-UPBLO, Ecole Polytechnique Fédérale de Lausanne (EPFL), 1015, Lausanne, Switzerland; [4]VIB Center for Medical Biotechnology, Department of Biomolecular Medicine, Ghent University, Ghent, Belgium.

SUPPLEMENTARY INFORMATION LEGENDS

Figure S1: Loss of importins does not affect resting localisation of SIRT2 (A) Graphs show nuclear: whole cell intensity ratio of SIRT2-GFP. Each data point represents a single cell across 3 independent experiments. **(B)** Western blot confirmation of importin siRNA-knockdown (left). HeLa cells were transfected with the indicated siRNAs and incubated for 48 hours. Immunoblots were performed on cell lysates probed for stated individual importins and a combination of KPNA2, TNPO1 and IPO7 (X3). Calculated percentage knockdown (KD) of individual siRNAs and combined X3 transfection. Uncropped blots are presented in Supplementary S3.

Figure S2: SIRT2:importin interactions are unaltered by cell type or tag position. (A) GFP, $SIRT2^{FL}$ or truncated $SIRT2^{1\text{-}356}$ were immunoprecipitated for 1 hr from HEK293T cells. Cell lysates (INPUT) and IP fractions were immunoblotted for with antibodies against GFP and KPNA2, IPO7 and TNPO1 Data are representative of 2 experiments**.** Full-length blots are presented in Supplementary S2. **(B)** mCherry, mCherry-$SIRT2^{FL}$ or truncated mCherry-$SIRT2^{1\text{-}356}$ were immunoprecipitated for 1 hr from HeLa cells. Cell lysates (INPUT) and IP fractions were visualised by stain-free gel imaging or were immunoblotted for with antibodies against KPNA2, IPO7 and TNPO1. Data are representative of 3 experiments**.** Full-length blots are presented in Supplementary S2. Full-length blots are presented in Supplementary S2.

Figure S3: Uncropped western blot images. All western blots which appear in article are uncropped and referenced with the figure to which they relate to.

Movie S1: Live microscopy of SIRT2^{FL} and SIRT2$^{1\text{-}356}$ following LMB treatment.

Cells expressing SIRT2^{FL} (green) or SIRT2$^{1\text{-}356}$ (Orange) were treated with 20 nM LMB and imaged at 5-minute intervals.

Table S1: List of human proteins identified and quantified by LC-MS/MS after co-immunoprecipitation of SIRT2-GFP and GFP (control). Proteins are sorted on descending SIRT2-GFP / GFP ratio (column D). Columns from left to right contain the Uniprot protein accession, gene name and protein name. The following columns show the protein fold change (in log2), -log p-value and statistical significance calculated by the Perseus t-test used for pair wise comparison of the SIRT2-GFP sample with the GFP control sample, followed by an indication whether the protein was identified as SIRT2 interactor without (this experiment) or with fractionation into the cytosolic, nuclear or chromatin fraction (independent experiment, data not shown). Finally, for each protein the number of identified MS/MS spectra, the number of identified peptides, the sequence coverage, the Andromeda score, the MaxQuant majority protein ID and the LFQ intensity values in each replicate sample are indicated.

Table S2: Gene ontology analysis of SIRT2 interactome. PANTHER Overrepresentation Test of significantly enriched SIRT2 interactors. Test used all *Homo sapiens* genes as reference list with Fisher's Exact.

Figure S1

A

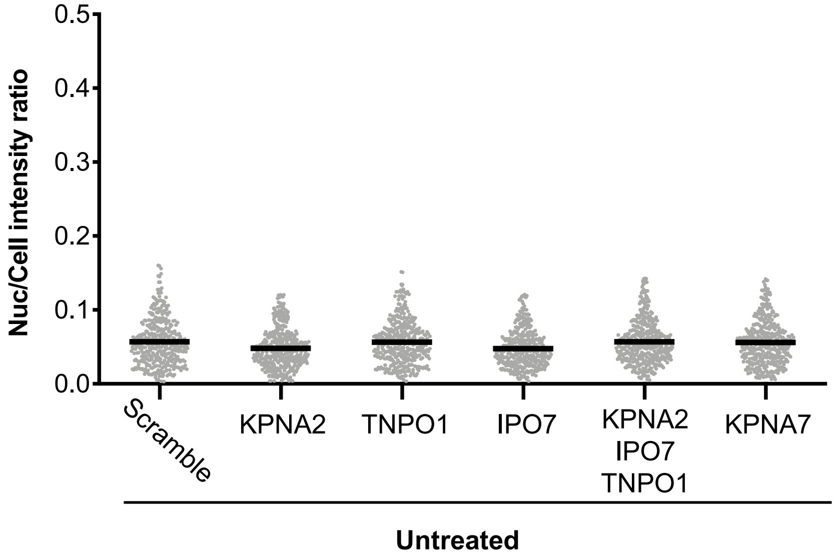

B

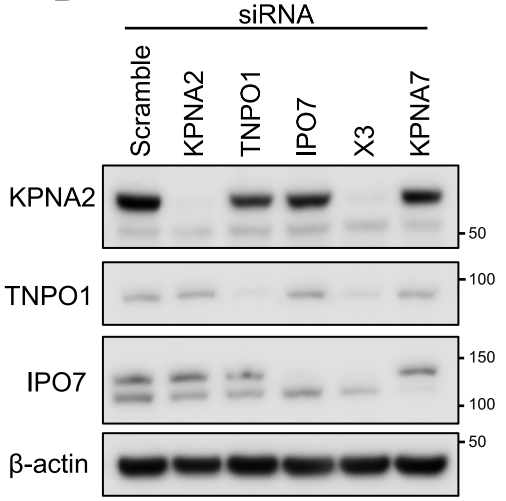

	% KD efficiency	
**siRNA**	Single	X3
**IPO7**	99.94	99.7
**KPNA2**	99.58	95.33
**TNPO1**	85.97	69.01

Figure S1: Loss of importins does not affect resting localisation of SIRT2

(A) Graphs show nuclear: whole cell intensity ratio of SIRT2-GFP. Each data point represents a single cell across 3 independent experiments.

(B) Western blot confirmation of importin siRNA-knockdown (left). HeLa cells were transfected with the indicated siRNAs and incubated for 48 hours. Immunoblots were performed on cell lysates probed for stated individual importins and a combination of KPNA2, TNPO1 and IPO7 (X3). Calculated percentage knockdown (KD) of individual siRNAs and combined X3 transfection. Uncropped blots are presented in Supplementary S3.

Figure S2

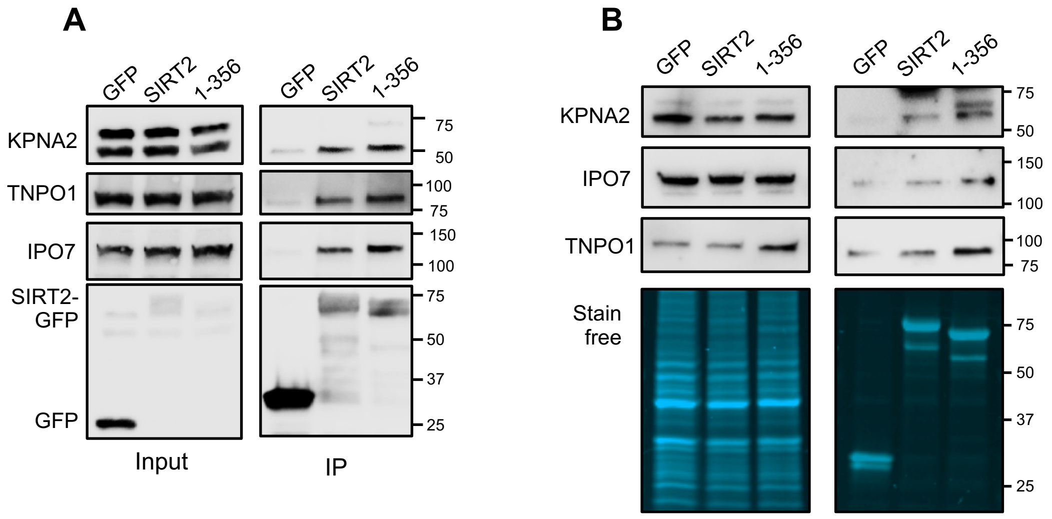

Figure S2: SIRT2:importin interactions are unaltered by cell type or tag position.

(A) GFP, SIRT2^{FL} or truncated SIRT2$^{1\text{-}356}$ were immunoprecipitated for 1 hr from HEK293T cells. Cell lysates (INPUT) and IP fractions were immunoblotted for with antibodies against GFP and KPNA2, IPO7 and TNPO1 Data are representative of 2 experiments**.** Full-length blots are presented in Supplementary S2.

(B) mCherry, mCherry-SIRT2^{FL} or truncated mCherry-SIRT2$^{1\text{-}356}$ were immunoprecipitated for 1 hr from HeLa cells. Cell lysates (INPUT) and IP fractions were visualised by stain-free gel imaging or were immunoblotted for with antibodies against KPNA2, IPO7 and TNPO1. Data are representative of 3 experiments**.** Full-length blots are presented in Supplementary S2.

Full-length blots are presented in Supplementary S2.

Figure S3

Relating to Figure 2A:

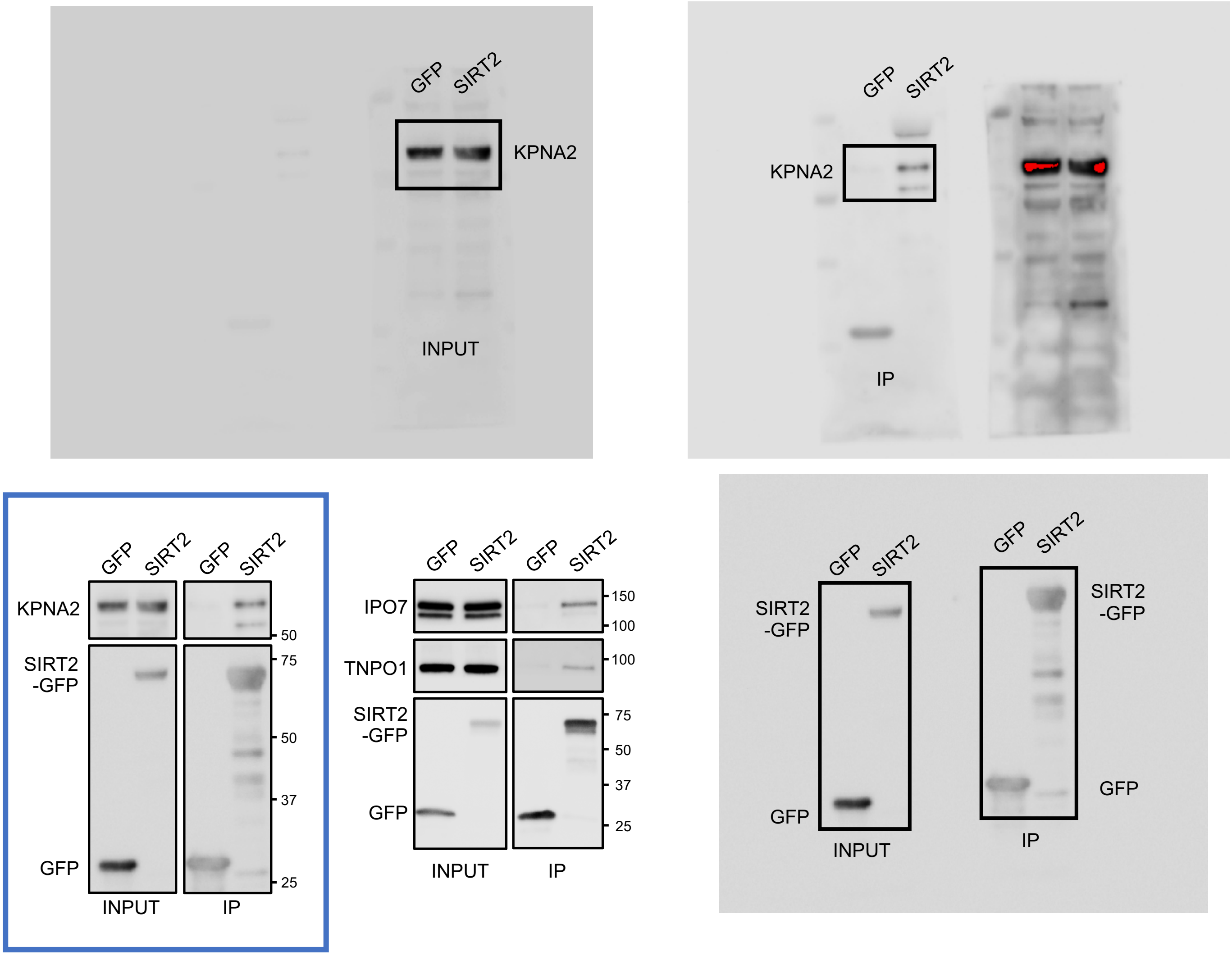

Relating to Figure 2A:

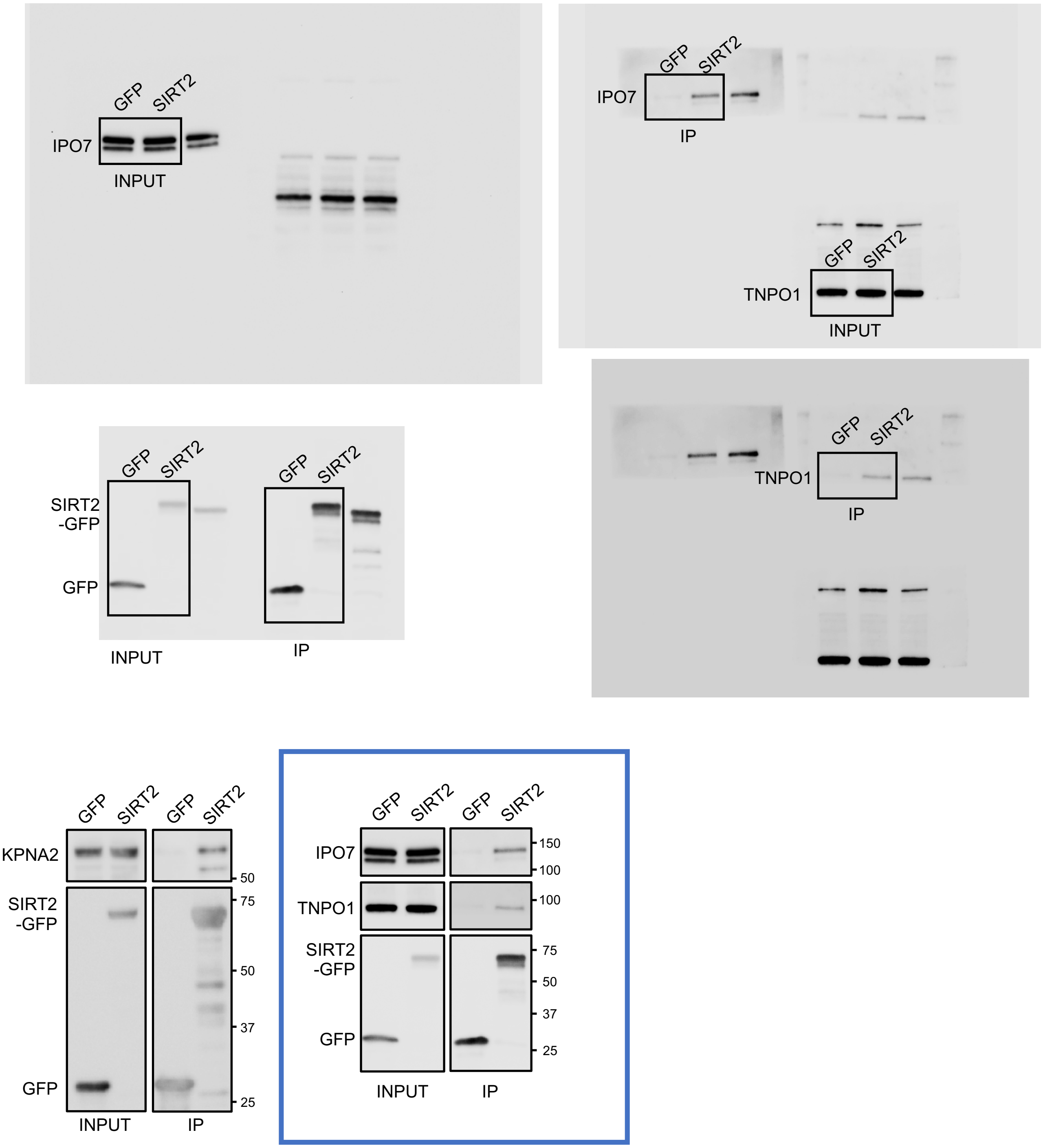

Relating to Figure 2B:

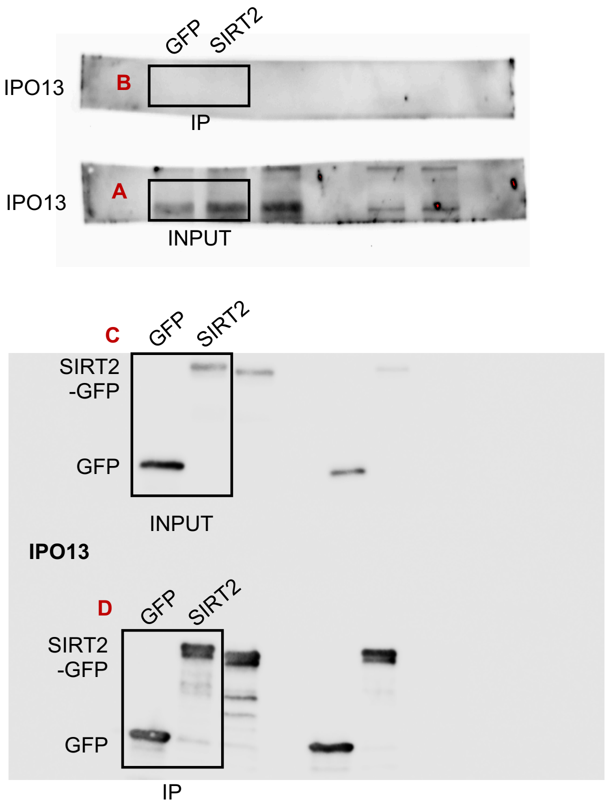

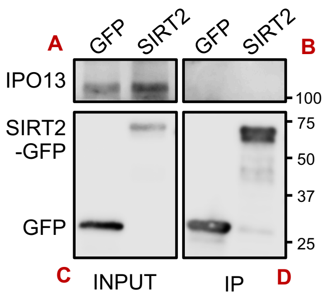

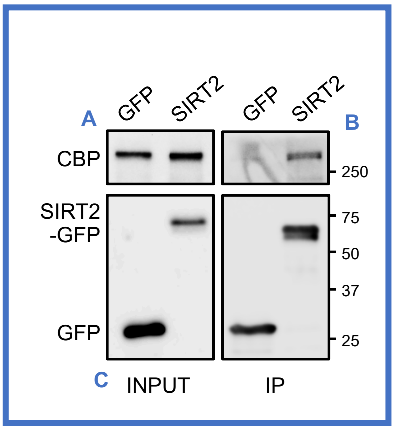

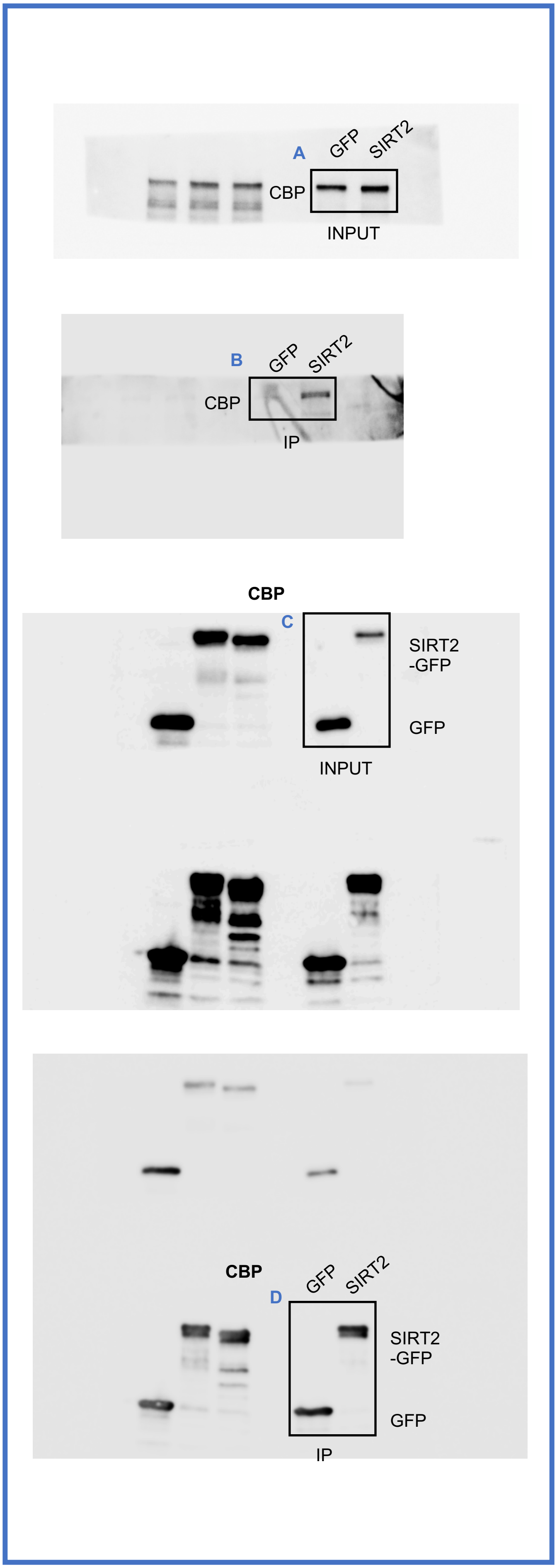

Relating to Figure 4B:

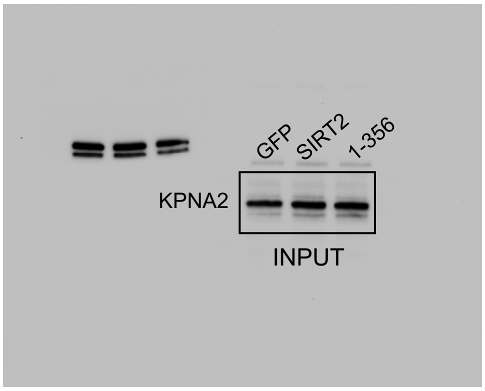

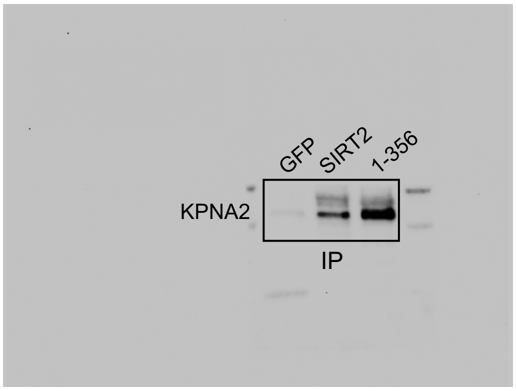

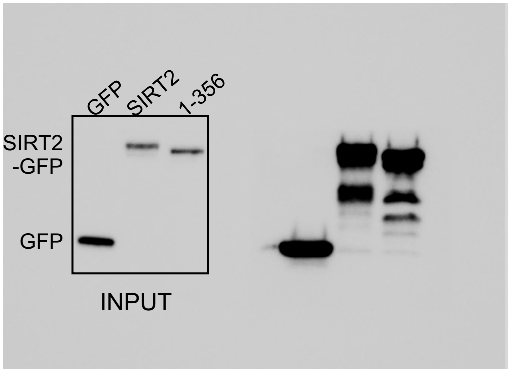

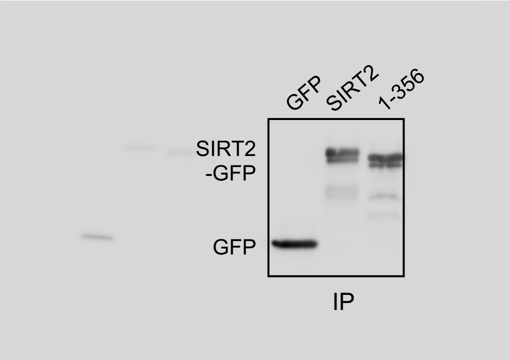

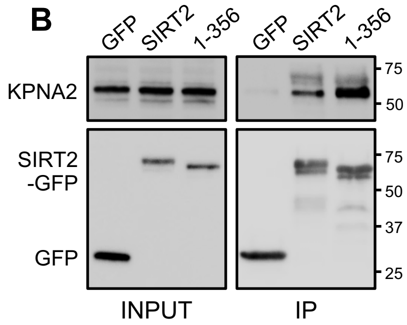

Relating to Figure 4C:

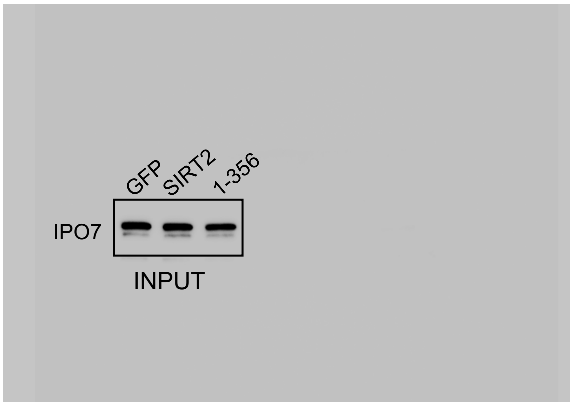

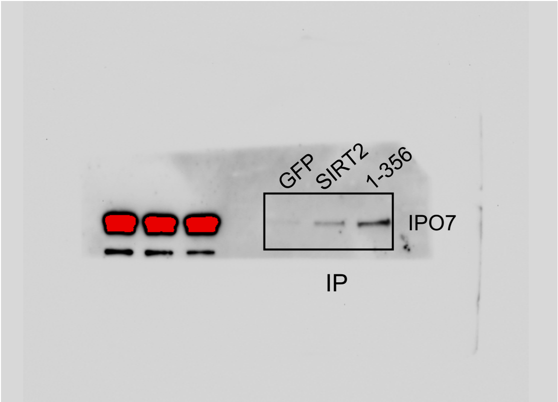

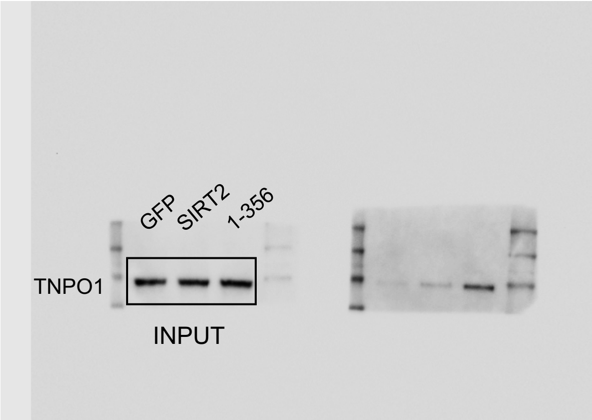

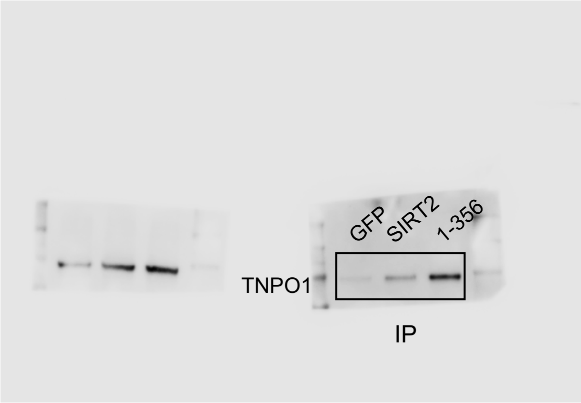

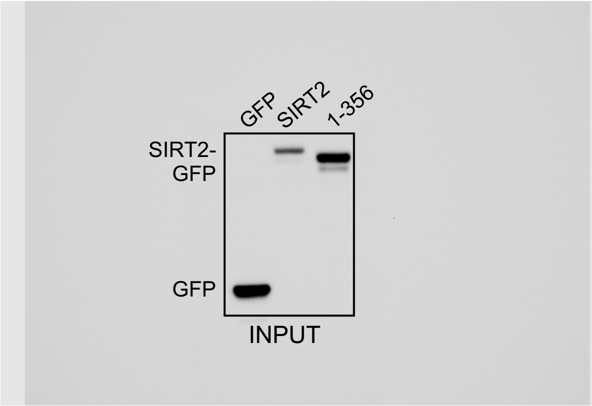

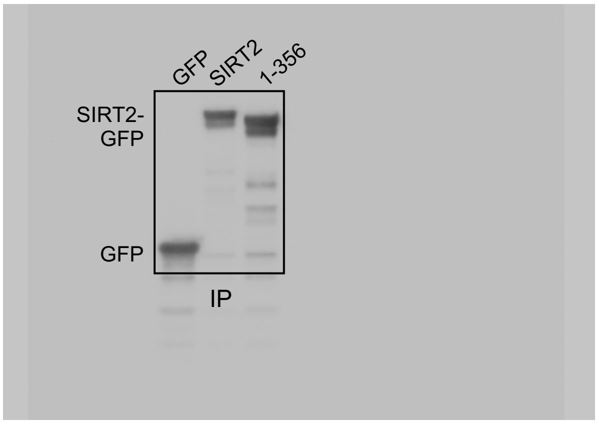

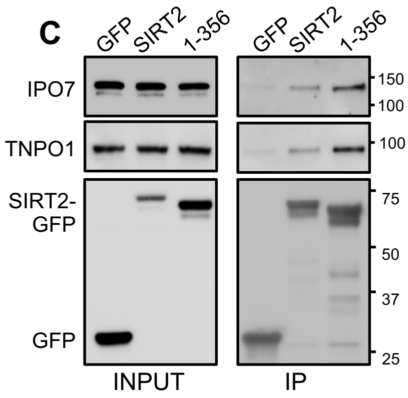

Relating to Figure 4D:

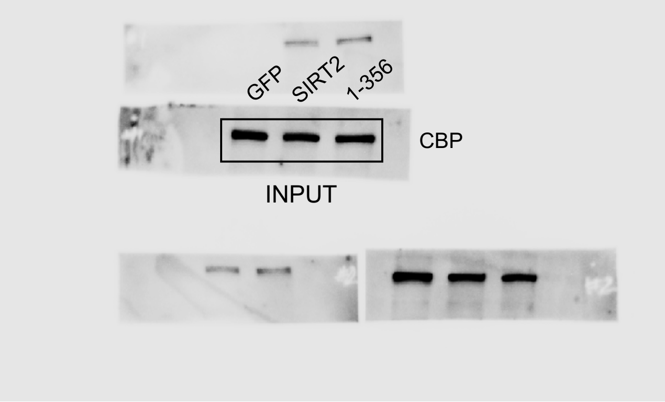

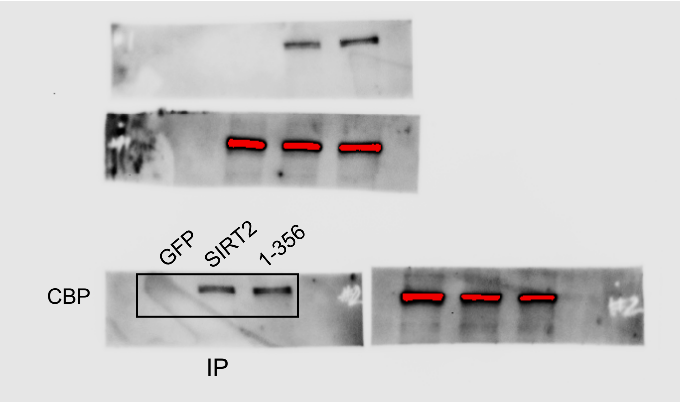

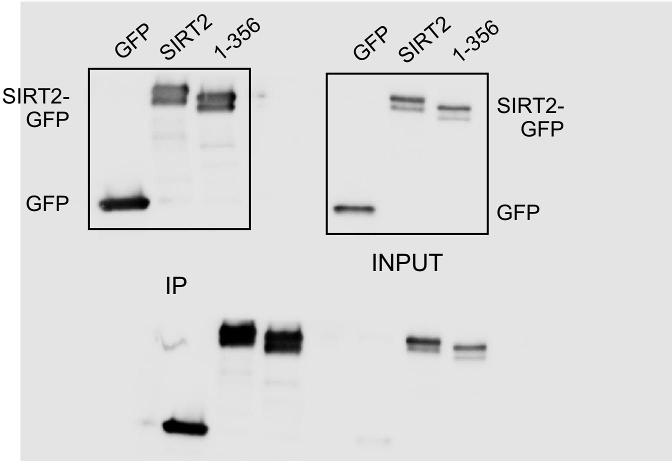

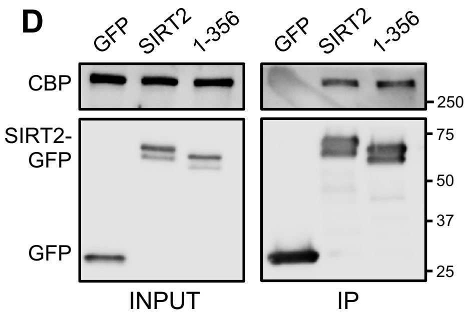

Relating to Figure 5A:

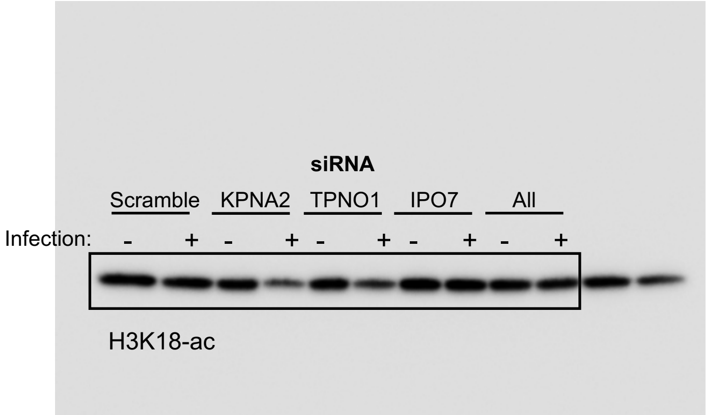

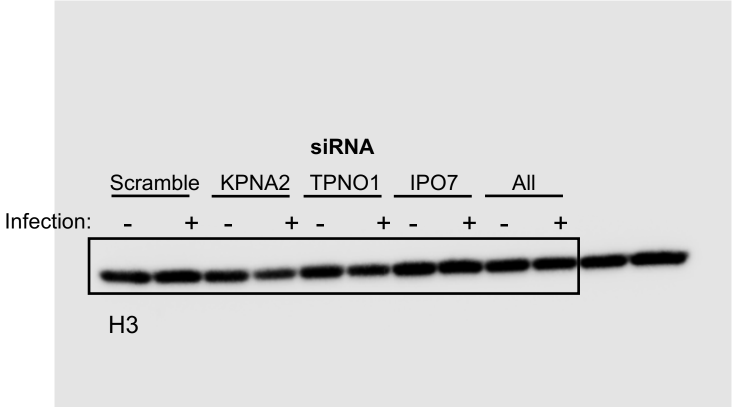

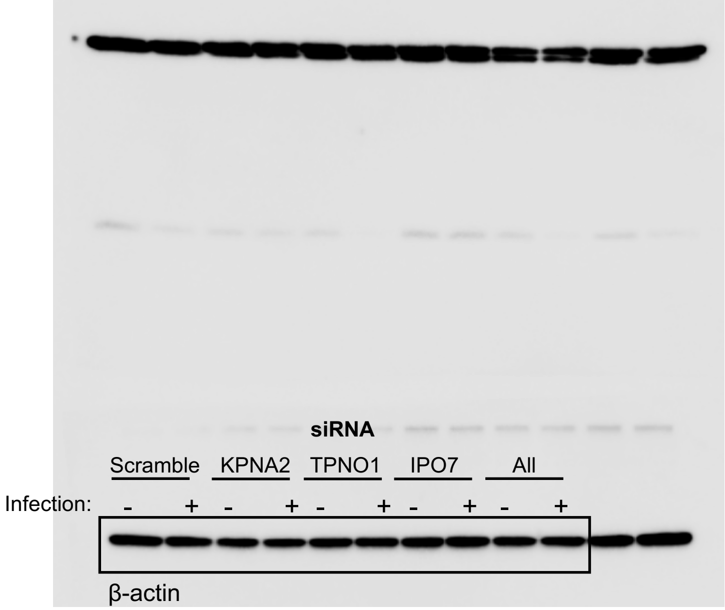

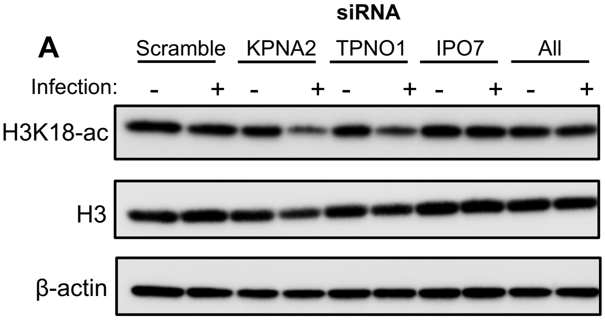

Relating to Figure S1:

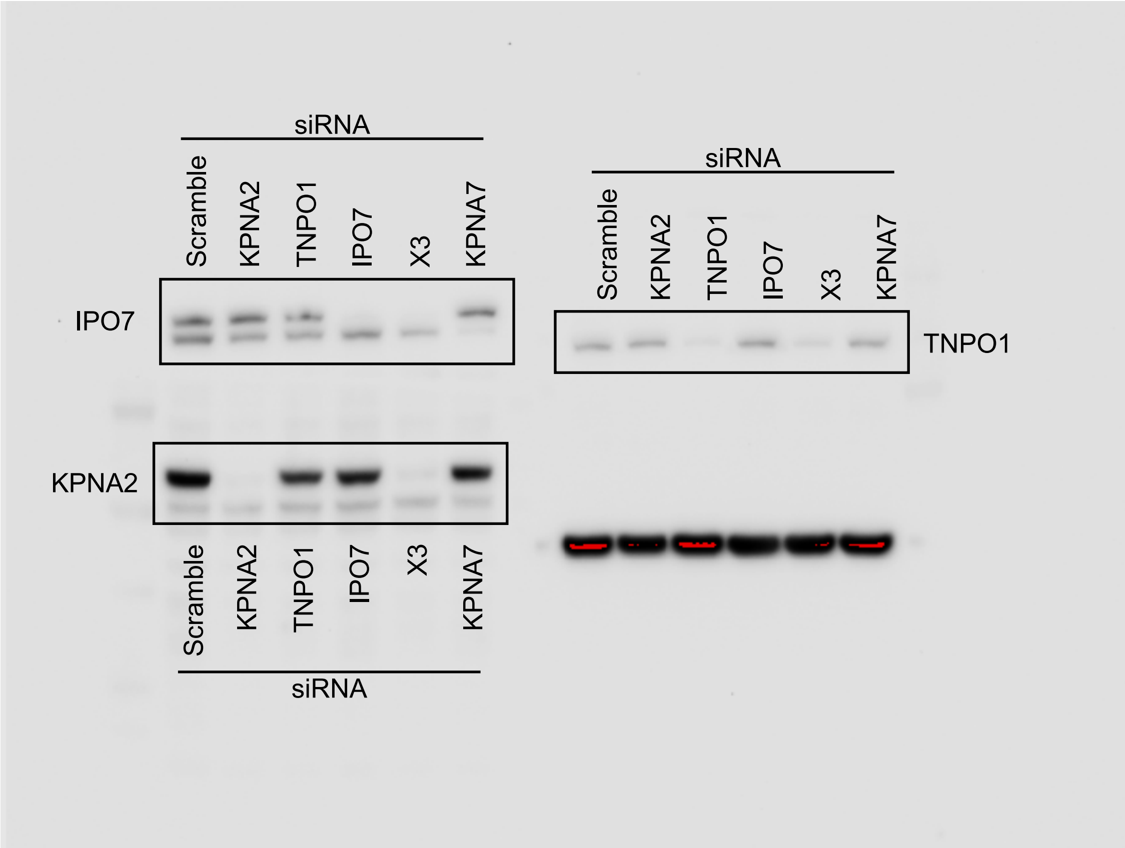

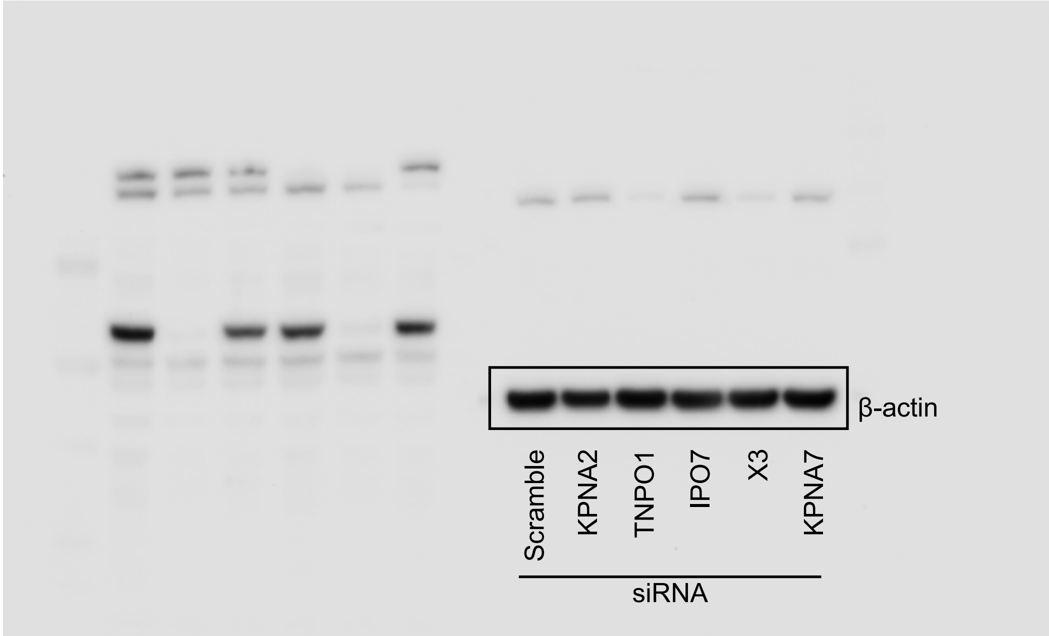

Relating to Figure S2A:

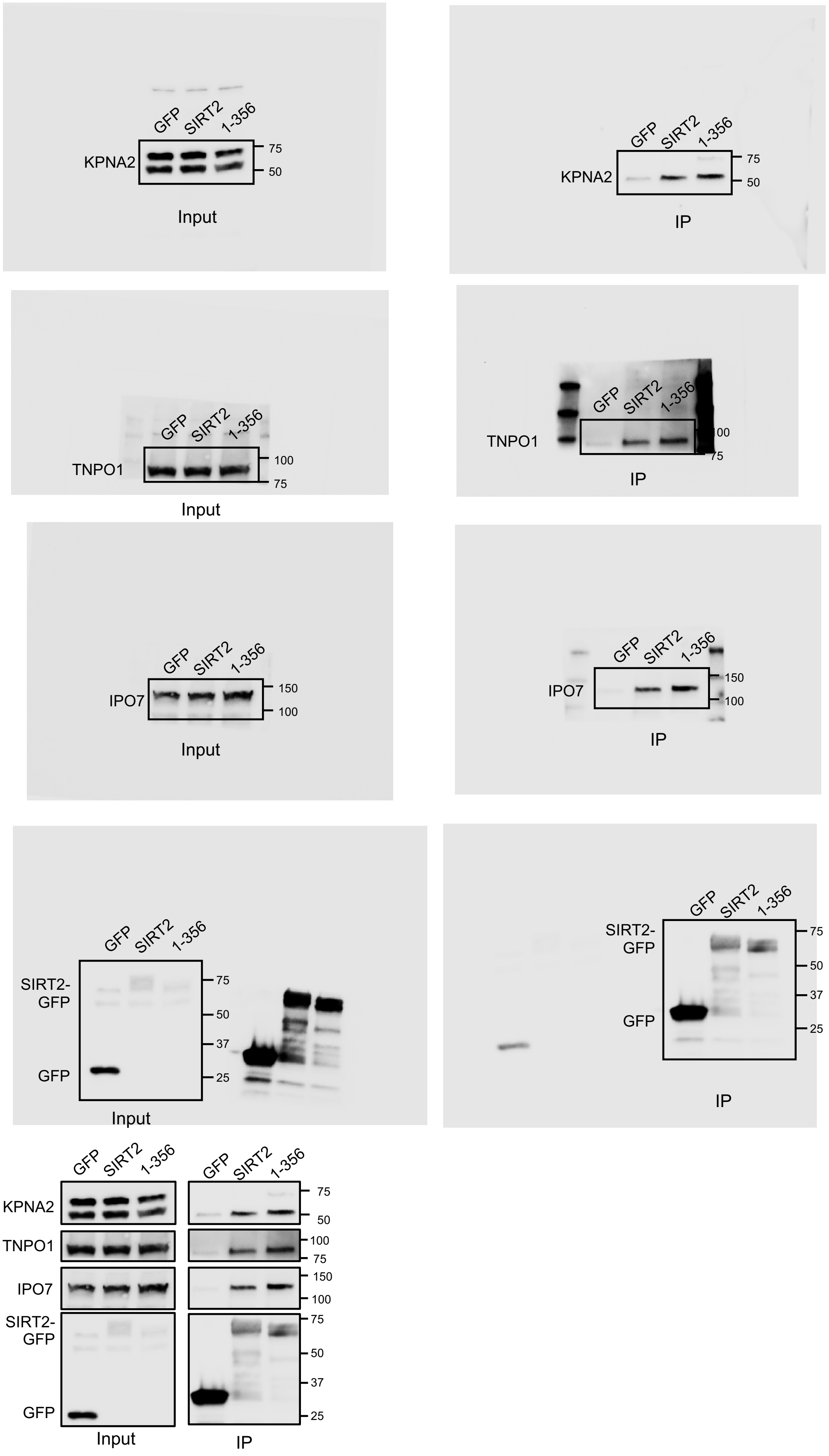

Relating to Figure S2B:

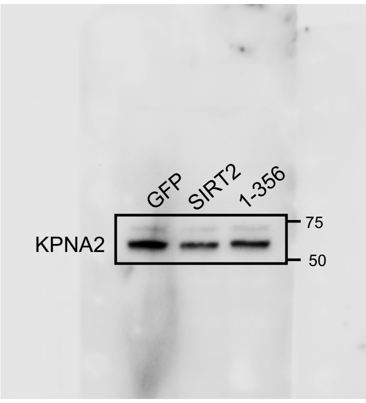

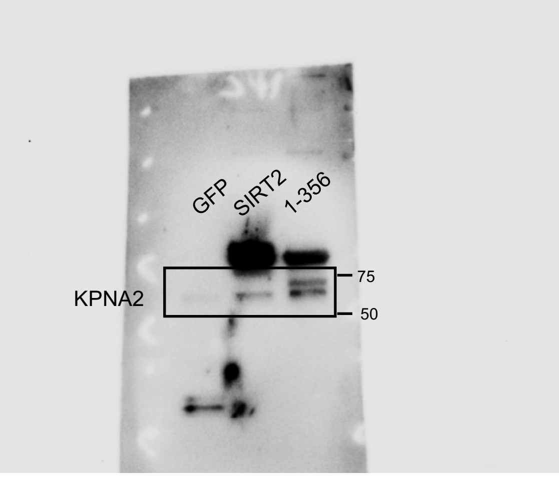

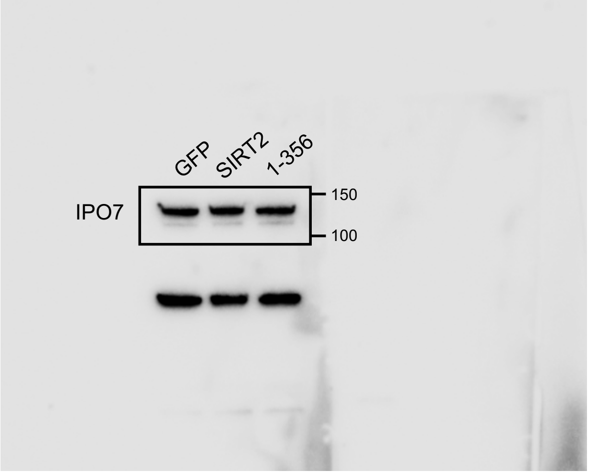

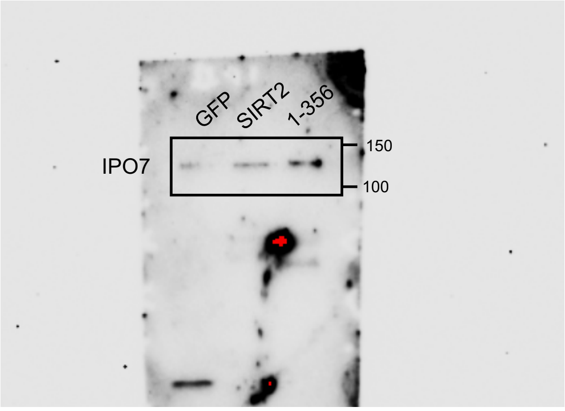

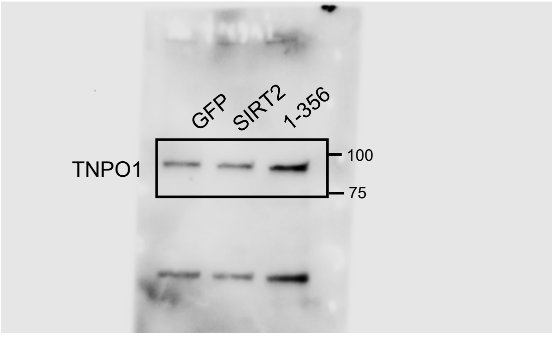

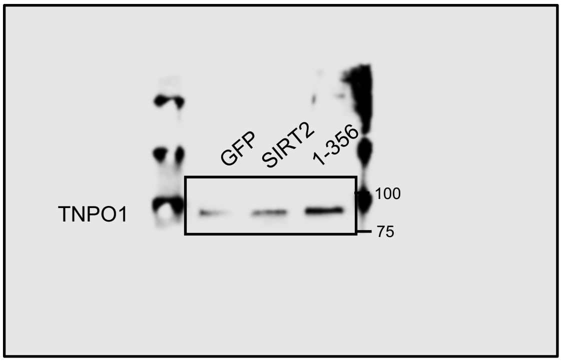

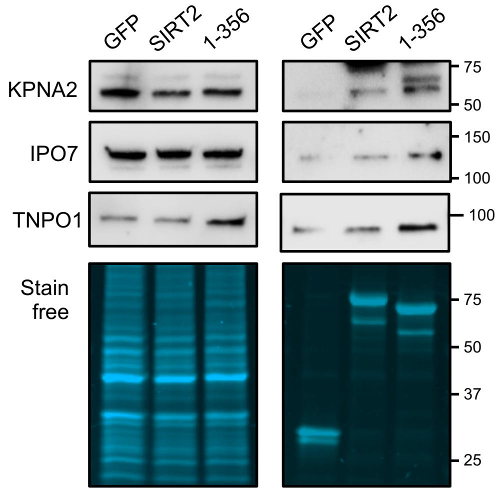

Relating to Figure S2B:

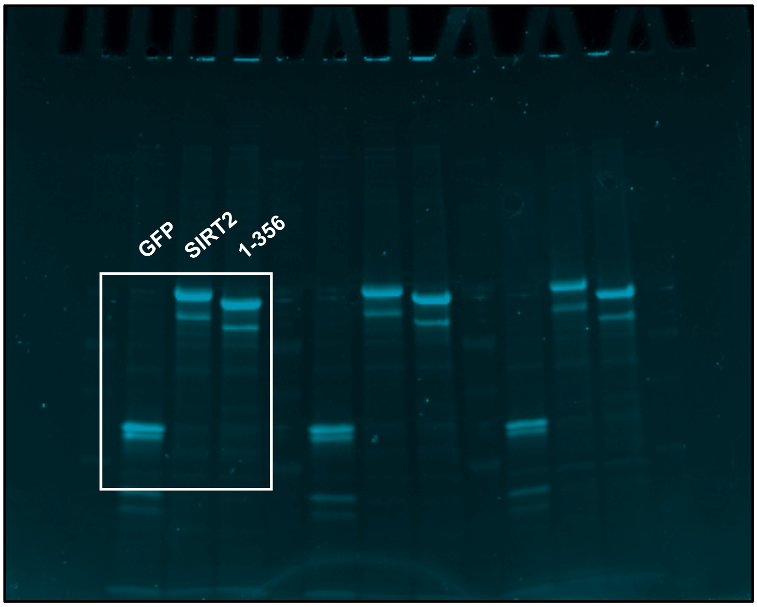

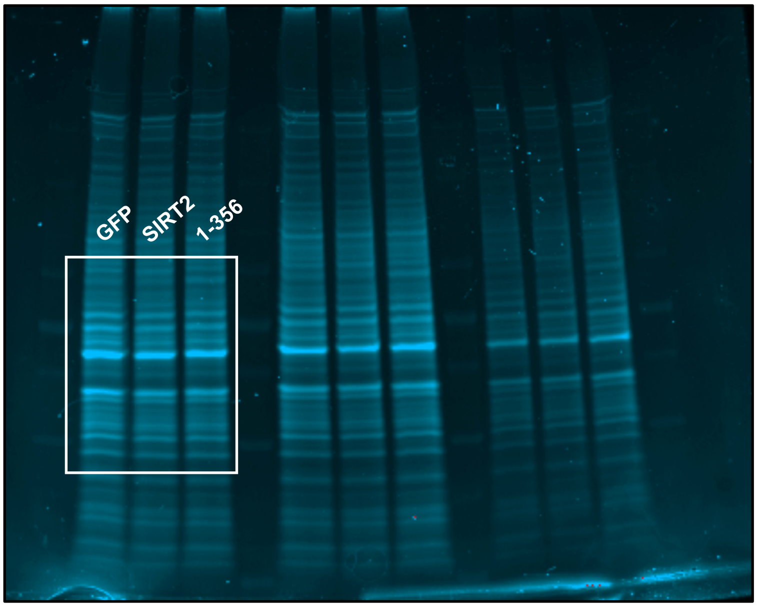

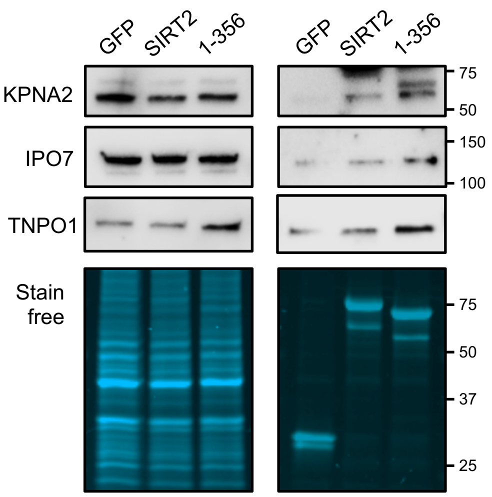

Supplement: Supplementary file 1 — Supplemental Information. [file 41598_2020_58397_MOESM1_ESM.pdf]
